# Comparing open surgical, SELDINGER’S technique with surgical isolation of the vein and ultrasound guided techniques for jugular central line insertion in infants: a randomized clinical trial

**DOI:** 10.1186/s12893-025-02988-5

**Published:** 2025-07-03

**Authors:** Mohamed Mahmoud Salah Eldin, Sherif Mohamed K. Shehata, Mohamed Ali Shehata, Ahmed Abdelmohimen Elhaddad

**Affiliations:** 1 Pediatric Surgery Department, Faculty of Medicine, Tanta University, Tanta, Egypt; 2 Pediatric Surgery Department, Faculty of Medicine, Tanta University Hospital, Tanta, Egypt

**Keywords:** Open Surgical, SELDINGER’S technique with surgical isolation of the vein, Percutaneous Ultrasound Techniques in Jugular, Central Line, Infants

## Abstract

**Background:**

Centrally Inserted Central Catheter (CICC) placing procedure is challenging in the pediatric population, especially in newborns and infants, leading to lower success and higher complication rates than in adults. The aim of this study was to compare three approaches: open technique, SELDINGER’S technique with surgical isolation of the vein, and percutaneous ultrasound-guided CICC insertion for central line insertion in infancy as regards safety, success of cannulation, technique time, and preservation of the patency of the internal jugular vein (IJV).

**Methods:**

This prospective randomized cohort study was conducted after approval of the Ethical Committee of Tanta University Hospital with approval code: 36264MS38/1/23 (clinical trial ID: NCT06862492 and date: 03/05/2025). This study adheres to CONSORT guidelines. This study included 105 infants in need of CVC insertion over a period of 6 months. They were randomly allocated into three equal groups; group A underwent CICC insertion using the open surgical technique, group B underwent SELDINGER’S technique with surgical isolation of the vein, and group C underwent percutaneous ultrasound-guided CICC insertion.

**Results:**

Patency was significantly higher in SELDINGER’S technique with surgical isolation of the vein and percutaneous ultrasound-guided techniques compared to the open surgical technique (*P* = 0.003, < 0.001). There was a significant negative correlation between patency of IJV and duration of CICC placement (*r* = -0.238, *P* = 0.010) and with the number of trials to success of the cannulation (*r* = -0.252, *P* = 0.006). The technique time was significantly shorter in the percutaneous ultrasound-guided technique compared to open surgical and SELDINGER’S technique with surgical isolation of the vein (*P* < 0.001, < 0.001). SELDINGER’S technique with surgical isolation of the vein was a significantly shorter technique time when compared to the open surgical technique (*P* < 0.001).

**Conclusions:**

US-guided catheterization of the IJV shows more advantages in the form of a less time-consuming technique with a high first attempt and insertion success rate and fewer trials compared to CICC insertion using either open surgical technique or SELDINGER’S technique with surgical isolation of the vein.

**Trial registration:**

Current Controlled Trials NCT06862492 and date: 03/05/2025.

**Supplementary Information:**

The online version contains supplementary material available at 10.1186/s12893-025-02988-5.

## Background

Centrally inserted central catheterization (CICC) is an essential technique in the intensive care units (ICUs) for the administration of life-saving treatments, including total parenteral nutrition, nutritional support, and intravenous medication [1]. CICC insertion procedure is challenging in pediatric patients, especially in newborns and infants, leading to lower success and higher complication rates than in adults [2]. Sites commonly used for CICC in infants are the internal jugular, subclavian, and femoral veins [3]. The internal jugular vein (IJV) is often chosen because of its relatively larger size than the subclavian vein, lower risk of complications, and easy compressibility in case of bleeding. To facilitate CICC, ultrasound (US) guidance over anatomical guidance in closed techniques has been introduced, bringing increased success rates, decreased catheterization times, and reduced complications [4].

Open surgical insertion is a standard method of tunneled catheter implantation in the past, but the percutaneous approach has recently gained more popularity [5]. The SELDINGER’S technique with surgical isolation of the vein insertion technique is a catheter over guide-wire technique, based on the original Seldinger’s technique, and has not been widely adopted in ICUs [6]. However, the SELDINGER’S technique with surgical isolation of the vein technique has been successfully introduced into the ICU, and it is now used as the only for peripherally inserted central venous catheter (PICCs) insertion [7].

CICCs have become a mandatory part of clinical management in a variety of clinical circumstances in pediatric age groups. It allows resuscitation for intravascular fluid depletion and access for vasoactive medications and antibiotics, and it provides a means for hemodynamic monitoring and pacing [8]. The US-guided closed technique is the updated use for insertion of IJV catheterization because it can both increase the success rate and decrease the complications related to CICCs placement [9].

The National Institute for Clinical Excellence (NICE) has recommended the use of US guidance as the preferred method for closed insertion of a CICC into the IJV in children [10].

In low-income and middle-income countries, the rate of complications of central catheters can be higher because of multiple insufficiencies, such as the unavailability of ultrasounds and adequate venous access devices, the lack of experience, and nursing quality. Therefore, enhancing healthcare team outcomes by assessing complications and their risk factors, and using an interprofessional approach with regular feedback is mandatory to reduce catheter-related morbidity [11].

The aim of this study was to compare three approaches: open technique, SELDINGER’S technique with surgical isolation of the vein, and percutaneous ultrasound-guided CICC insertion of central line insertion in infancy as regards safety, success of cannulation, technique time, and preservation of the patency of the internal jugular vein, in a prospective way.

## Methods

This prospective randomized cohort study included 105 infants over a period of 6 months, from July 2023 to December 2023.

The study was conducted after approval of the Ethical Committee of Tanta University Hospital with approval code: 36264MS38/1/23. Informed consent was obtained from the patients’ parents before participating in this study. The research was carried out in accordance with the Declaration of Helsinki. The study adheres to CONSORT guidelines.

Inclusion criteria were patients with an age ranging from birth to two years, patients with benign conditions needed CICC in the IJV for medical or surgical causes such as emergency cases (as trauma, shock), intraoperative resuscitation, intraoperative replacement therapy, and several medical conditions).

Exclusion criteria were femoral or subclavian CICCs, patients with thrombosed IJV, patients with previous CICC and those with malignant conditions.

### Randomization

This study is open-label. The participants were randomized using a computer-generated list of random numbers sealed in an opaque envelope and were randomly allocated into three equal groups on a scale of 1:1:1.

- Group A: included 35 patients who underwent CICC using an open surgical technique.
- Group B: included 35 patients who underwent CICC using SELDINGER’S technique with surgical isolation of the vein.
- Group C: included 35 patients who underwent percutaneous ultrasound-guided CICC.

All patients were subjected to full history taking, clinical examination, and laboratory investigations, including preoperative complete blood count (CBC) and coagulation profile (Prothrombin time (PT) normal range at birth (0–1 day): 13.5—20 s, 1–30 days: 12.5- 17 s, 1–6 months: 11.5–15 s and 6 months–2 years: 11–14 s, partial thromboplastin time (PTT/aPTT) normal range at birth (0–1 day): 39–90 s, 1–30 days: 35–70 s, 1–6 months: 28–55 s and 6 months–2 years: 25–45 s; international normalized ratio (INR) normal range at birth: 1.3–1.7, 1–30 days: 1.2–1.6, 1–6 months: 1.1–1.4 and 6 months–2 years: 0.9–1.2) [12].

Ultrasound (U/S) Doppler assessment was conducted for patency of the IJV 2 weeks after catheter removal.

### Technique

The patients who were operated on in the operative rooms underwent general anesthesia as follows: standard monitoring (oxygen saturation, electrocardiogram, non-invasive Blood Pressure), pre-oxygenation, and inhalational induction using sevoflurane till the IV line was established. Propofol (2.5 mg/kg), fentanyl (2 µg/kg) and atracurium (0.5 mg/kg) were used. The intubation was done using a cuffed endotracheal tube (ETT) suitable for age and weight, and its right position was confirmed by auscultation of bilateral equal breath sounds, then connected to the mechanical ventilator, and inhalational maintenance was provided using sevoflurane 2% (1 MAC).

The patients who were operated on in the NICU and PICU were sedated using midazolam (0.1–0.2 mg/kg bolus).

### Open surgical cut-down technique

The technique was performed following the descriptions of Farhadi et al. [13]. The infant was positioned in 30° (Trendelenburg's position), with a roll under his shoulders for neck extension, and rotated to the contralateral side of the surgical side to expose the incision site. Incision was done under sedation, and a pulse oximeter was used to monitor the oxygen saturation during the procedure.

**Fig. 1:**
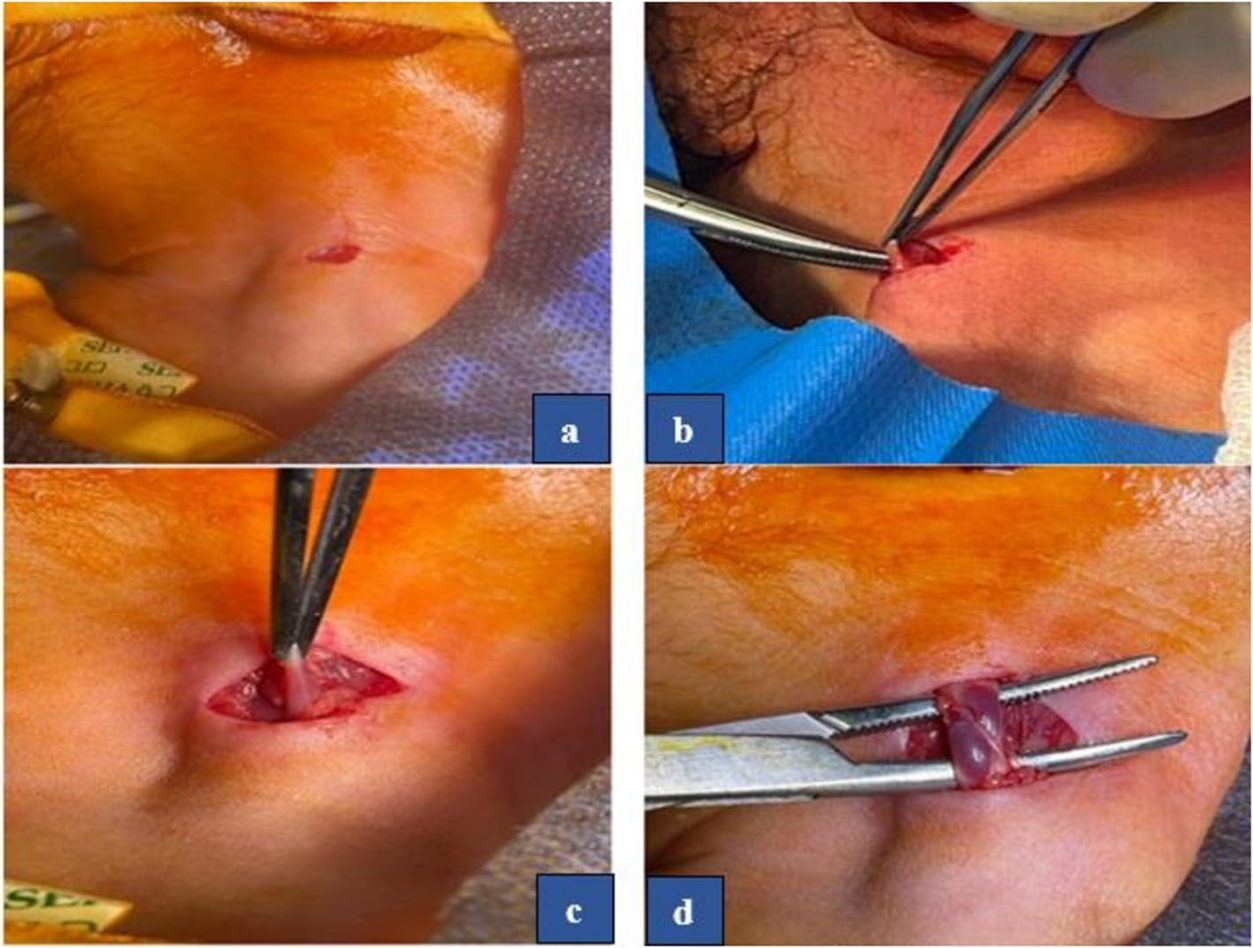
Panel of operative photographs showing the first steps of open CVC insertion: (**a**) a small transverse incision l cm was made on triangle which was bordered by the clavicle inferiorly and by the sternal and clavicular heads of the sternomastoid muscle medially and laterally, (**b**) blunt dissection to separate the two heads of the sternomastoid exposing the IJV, (**c**) identification of IJV from the internal carotid artery, (**d**) complete control of IJV

Under complete aseptic technique, a small transverse incision 1 cm was made on the triangle bordered by the clavicle inferiorly and by the sternal and clavicular heads of the sternomastoid muscle medially and laterally. With blunt dissection, we separate the two heads of the sternomastoid, exposing the IJV. Figure 1.

**Fig. 2:**
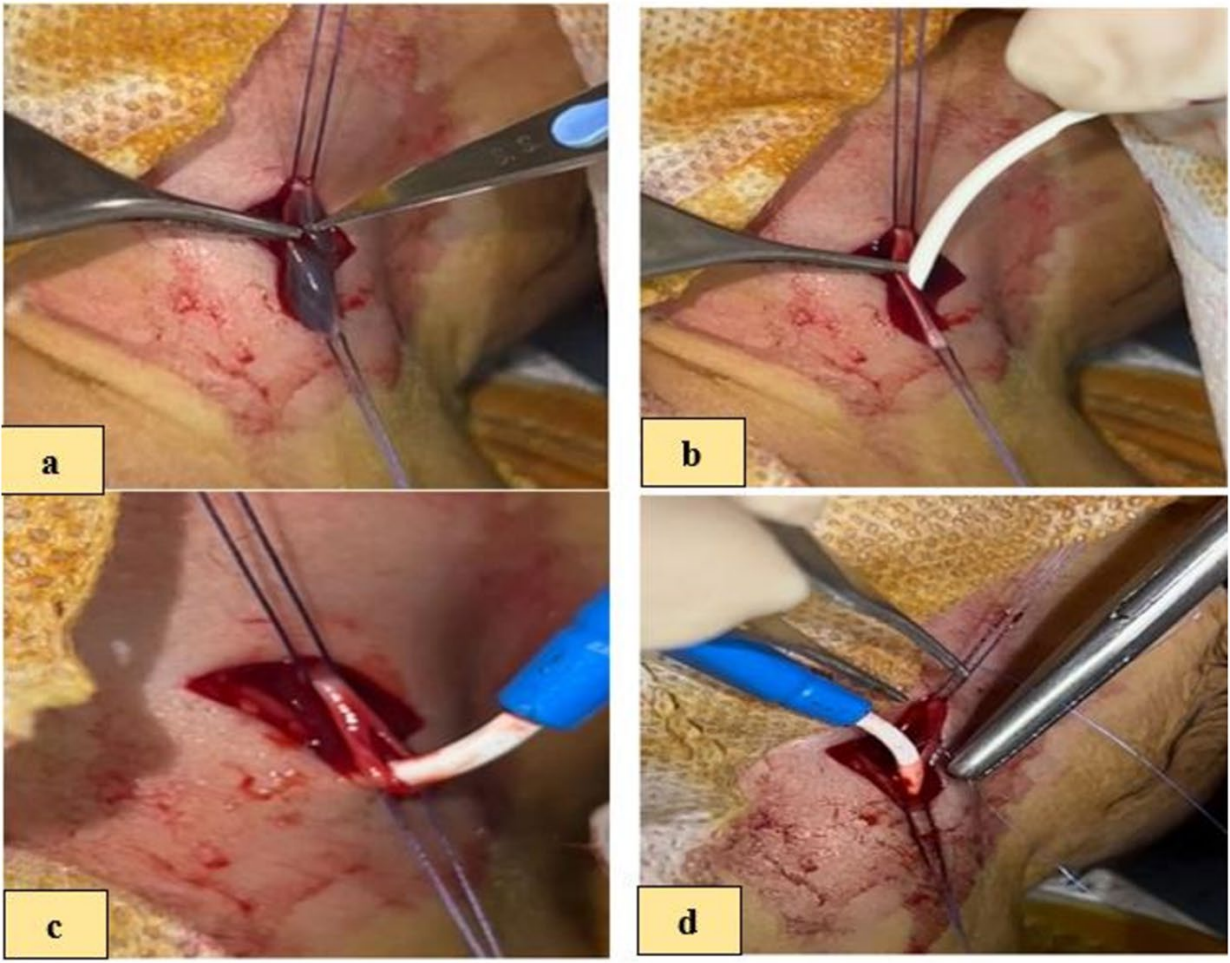
Panel of operative photographs showing the open venotomy technique of CVC insertion: (**a**) cut down of the IJV, (**b**) catheter insertion through the cut down defect, (**c**) the defect of IJV after catheter insertion, (**d**) the venotomies were repaired by 6/0 Polypropylene suture

Then, the IJV cutdown was performed, and a catheter was inserted through it. All IJV venotomies were repaired as needed by 6/0 Polypropylene (Prolene®) continuous suturing, and the wound was closed by absorbable polyglactin (Vicryl) suture. Figure 2.

### SELDINGER’S technique with surgical isolation of the vein

**Fig. 3:**
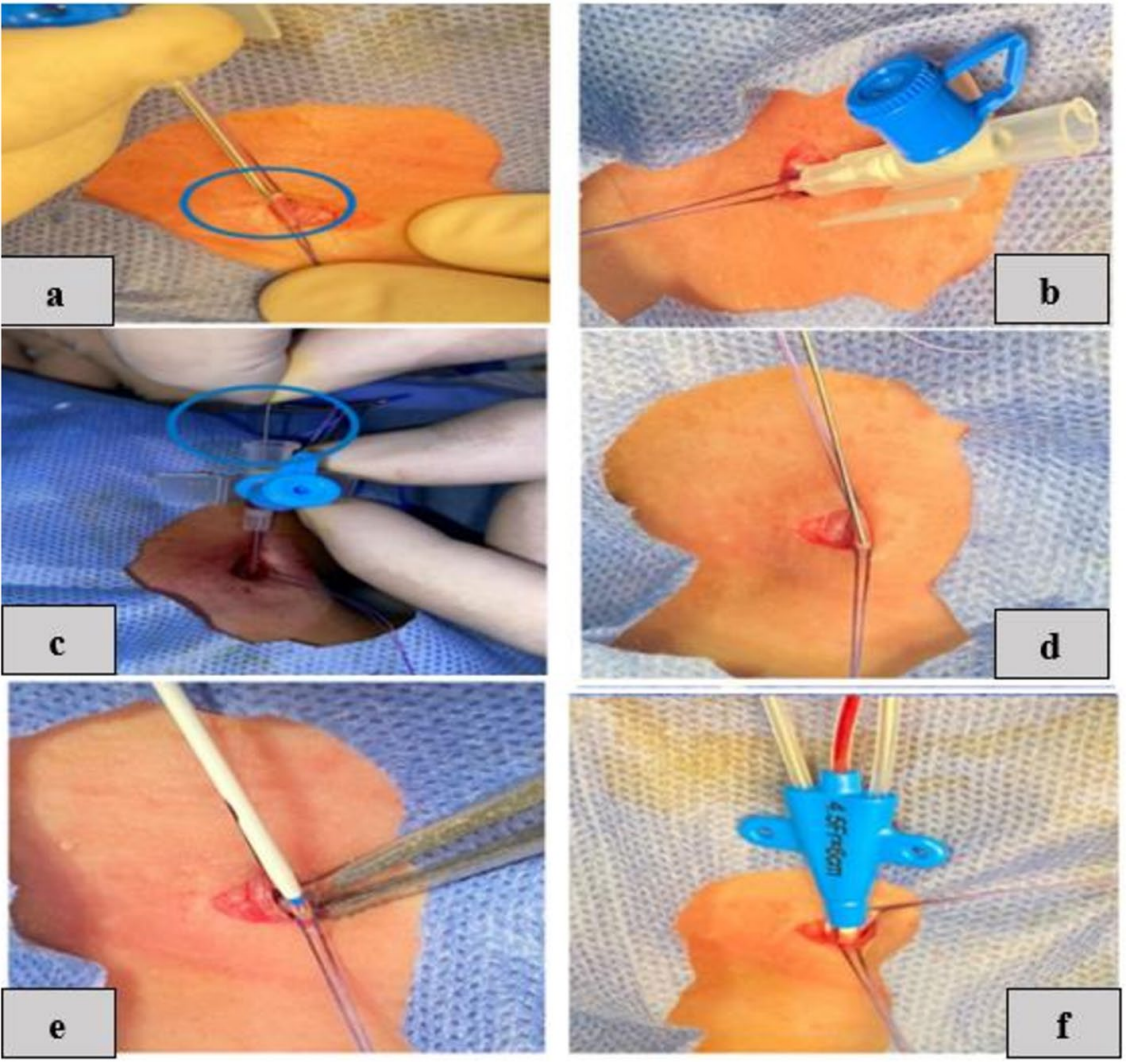
Panel of operative photographs showing the open SELDINGER’S technicque with surgical isolation of the vein technique of CVC insertion: (**a**) a 24-G. Cannula was carefully inserted directly to the IJV, (**b**) removal of the canula’ trocar, (**c**) guide-wire insertion through the cannula, (**d**) removal of the cannula leaving the guide-wire alone inside the vein, (**e**) catheter was passed and brought out through the guide-wire, (**f**) triple channels CVC with the main channel shows backflow of blood after removal of guide-wire

By using the same steps of the previous technique, after proximal and distal control of the vein, a 24-G. The cannula was carefully inserted directly into the IJV. The straighten J tip of guide-wire was inserted through the cannula. The catheter diameter was variable according to the vein size. Then, the cannula was removed. A size 4–5 French short length catheter was passed and brought out through the guide-wire, then the guide-wire was removed. The ratio between the diameter of the vein and the size of the catheter 1/3 is the ideal ratio to avoid thrombosis. The wound was closed after the correct catheter position and good haemostasis was obtained; the area was covered with a sterile dressing. Figure 3.

### Percutaneous US-guided central venous catheter insertion

**Fig. 4:**
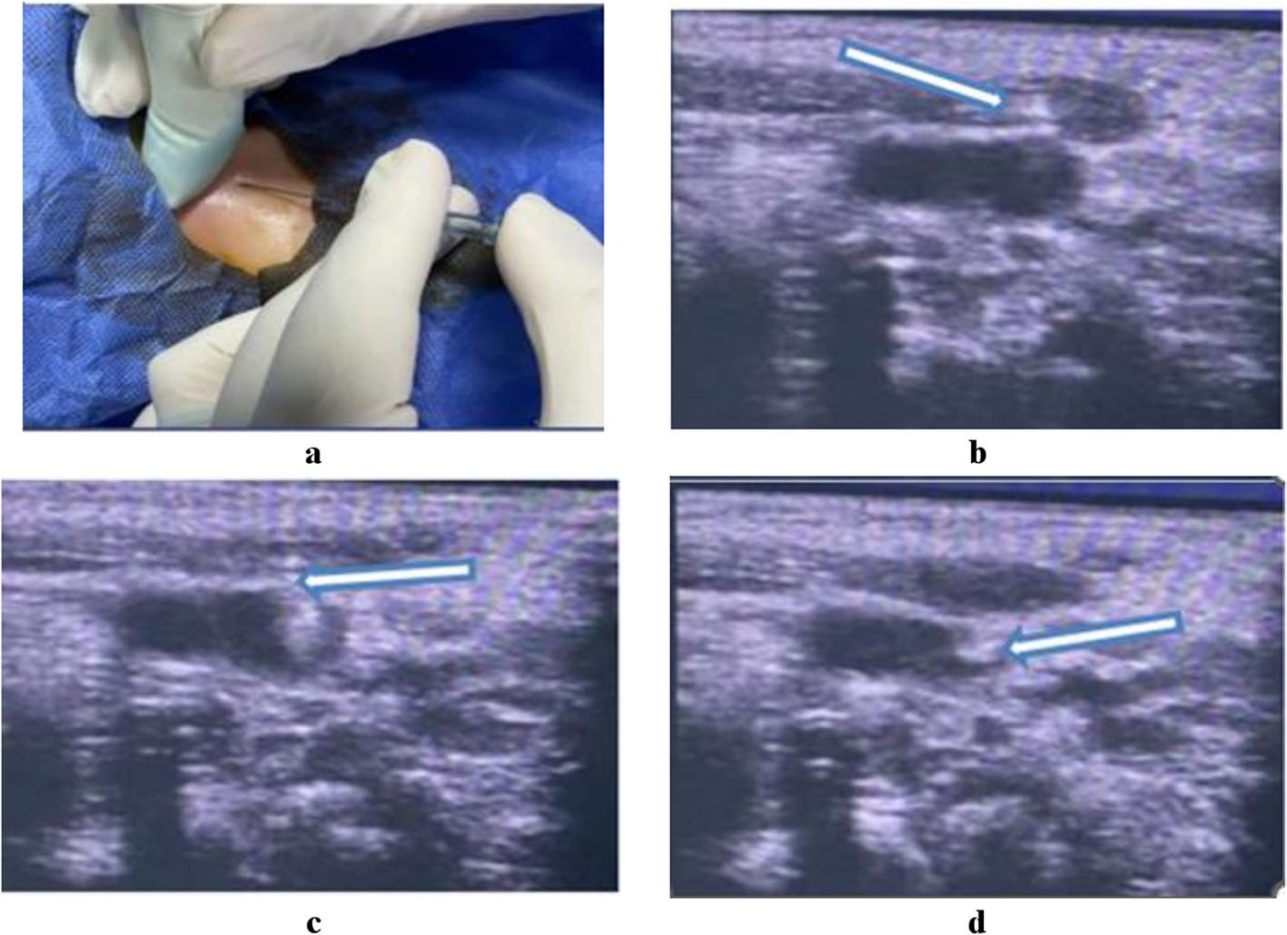
Panel of photographs showing percutaneous US guided central venous catheter insertion: (**a**) operative photograph showed U.S Guided puncturing by needle, (**b**) US photo where the filled arrow denotes needle outside the vein wall, (**c**) US photo where the filled arrow denotes needle penetrating the vein wall, (**d**) US photo where the filled arrow denotes needle inside the vein lumen

**Fig. 5:**
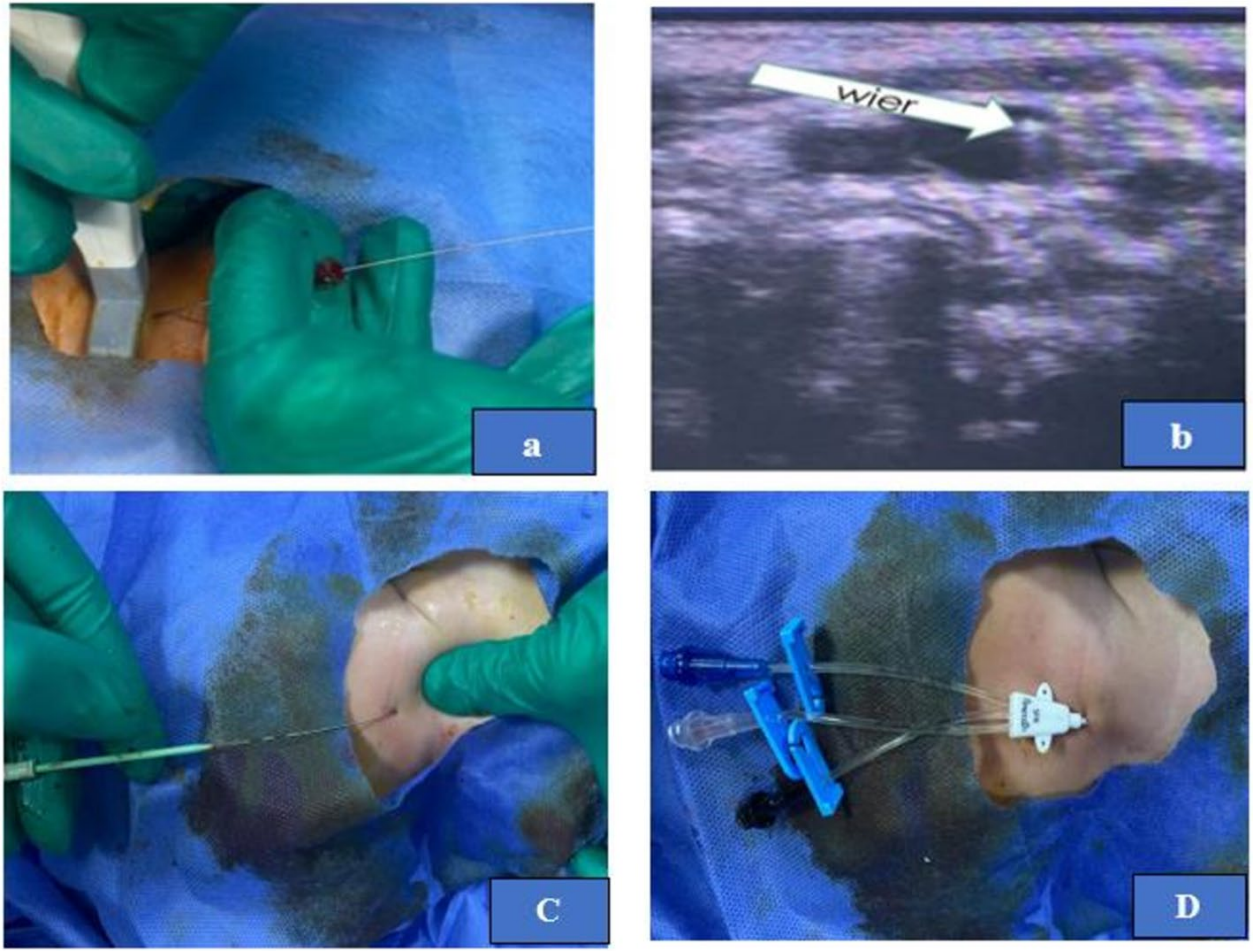
Panel of photographs showing percutaneous US guided central venous catheter insertion: (**a**) operative photograph showed insertion of the guide-wire into the needle under US guidance, (**b**) sonographic view where the filled arrow denotes the wire inside the vein, (**c**) insertion of skin and venous dilator over the wire then remove the dilator, (**d**) insertion of the catheter over the guide-wire inside the vein, then removal of the guide-wire

The insertion area was prepared as previously described. The ultrasound probe was connected to the US unit and focused with ultrasonic gel and wrapped in a sterile plastic sheath. By wrapping the transducer in a sterile sheath, the probe is placed perpendicular to the vein to obtain a view of the short axis of the vein, and standard US two-dimensional (2D) imaging was used to visualise the vein as a circle. Catheterization was performed under continuous dynamic observation of real-time 2D images. The catheter which we used in our study had a conical tip, but in the percutaneous technique we used the dilator. The insertion needle was advanced through the skin under US guidance into the IJV. A guide-wire was then placed through the needle into the vein, and the needle was removed. Then the catheter was inserted over the wire into the IJV. Figures 4and 5.

We used a superficial probe (Mindray, Shenzhen, China), linear array probe 7–15 MHz can show 2–7 cm depth and has large foot-print so used in large infants, or small linear “Hockey-stick” probe 6–15 MHz can show 2–5 cm depth and has small foot-print so used in small infants.

In all techniques, radiographic assessment by X-ray was performed after insertion of catheter at NICU and PICU to ensure catheter tip location and to detect the presence of pneumothorax, the optimal depth of catheter insertion was guided by the standard equation [length of insertion (cm) = 1.7 + (0.07 × height)], we never cut the catheter. If it is long, withdrawal of the catheter to outside was done and then an extension was applied and attached, after which. A 4/0 silk suture was placed on the skin to fix the line at the exit site. We didn’t use sutureless device, as it was unavailable in our centre. For flushing of the catheter, we used the old fashion approach, and we recommend using the normal saline instead of the heparinized, as no difference between both.

During the techniques, the pulse was monitored for the detection of arrhythmia or bradycardia, which might occur during the insertion of the guide-wire or the tip of the catheter.

### Doppler US assessment 2 weeks following removal of CICC

Doppler US was performed to assess patency of the jugular vein, evaluation of margins, regular changes in diameter with respiration or sniffing, flow assessment, and presence or absence of intraluminal filling defect was made, plus the diameter of CICC was measured.

The maintenance of the CICC was managed by experienced nurses according to a standardized protocol. Also, nurses provide a report in case of the incidence of any complications, additionally, the catheter can be removed by experienced nurses after the doctor’s approval [14].

Success of cannulation was defined as no intraoperative complications as pneumothorax, hemothorax, or rupture of a vein. If the cases were polytrauma and needed multiple punctures (more than 2) were excluded.

The criteria for first vs. second attempt success was that First attempt = first puncture Failed first attempt, we need second trial to puncture to the vein.

The primary outcome: was the assessment of the technique time.

The secondary outcome: was evaluation of successful cannulation, patency of the IJV, and a totally thrombosed IJV.

### Sample size calculation

The sample size calculation was done by G*Power 3.1.9.2 (Universität Kiel, Germany). We performed a pilot study (5 cases in each group), and we found that the mean of technique time (the primary outcome) was 16.32 min in group A, 13.57 min in group B, and 9.5 min in group C. The sample size was based on the following considerations: 0.99 effect size, 95% confidence limit, 95% power of the study, group ratio 1:1:1, and 12 cases were added to each group to overcome dropout. Therefore, we recruited 105 patients (35 patients in each group).

### Statistical analysis

Statistical analysis was done using Statistical Package for the Social Sciences (SPSS) v27 (IBM©, Armonk, IL, USA). The Shapiro-Wilks test and histograms were used to evaluate the normality of the distribution of data. Quantitative parametric data were presented as mean and standard deviation (SD) and were analysed by one-way ANOVA (F) test with post hoc test (Tukey). Levene's Test for Homogeneity of Variances To ensure the validity of the ANOVA assumptions, Levene's test was conducted to assess the homogeneity of variances across groups. This test determines whether the assumption of equal variances holds, which is crucial for the reliability of ANOVA results. Quantitative non-parametric data were presented as median and interquartile range (IQR) and were analysed using the Kruskal–Wallis test and/or the Mann–Whitney test to compare each group. Qualitative variables were presented as frequency and percentage (%) and were analysed utilizing the Chi-square test. A two-tailed probability (*P*) value < 0.05 was considered statistically significant. A 95% confidence interval (CI) of the mean was represented, and it is a range with an upper and lower number calculated from a sample.

## Results

**Fig. 6:**
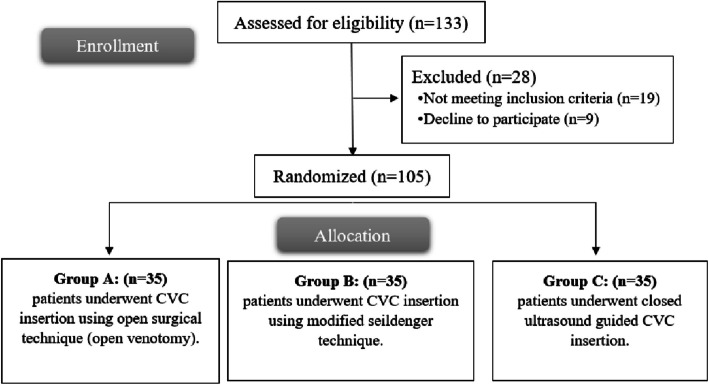
CONSORT flowchart of the enrolled patients

In this study, 133 patients were assessed for eligibility; 19 patients did not meet the criteria, and 9 patients refused to participate in the study. The remaining 105 patients were randomly allocated into 3 equal groups (35 patients in each group). All allocated patients were followed up and analysed statistically. Figure 6.

There was an insignificant difference among the studied groups regarding the demographic data or the indication of insertion, as depicted in Table 1. Fifty-two cases done bedside in the NICU, 13 cases in the PICU, and 40 cases in the operative rooms with all three techniques.

**Table 1:** Demographic data and indications of insertion of the studied groups

		Group A (*n*=35)		Group B (*n*=35)		Group C (*n*=35)		Test of sig		95%CI		*P* value
Age (days)	Mean ± SD	36.2 ± 32.58		47.11 ± 74.35		49.34 ± 80.97		KW = 5.995		28.757 to 37.176		0.675
	Median (IQR)	35 (13- 50)		34 (16.5–47)		35 (17.5–120)						
Sex	Male	19 (54.28%)		18 (51.42%)		20 (57.14%)		X2 = 0.2303		0.6084 to 1.4751		0.549
	Female	16 (45.71%)		17 (48.57%)		15 (42.85%)						
Weight (Kg)	Mean ± SD	4.39 ± 1.65		4.55 ± 1.75		4.32 ± 1.32		F = 0.1939		3.823 to 4.774		0.853
	Median (IQR)	2.5 (2.02–3.15)		2.8 (2.4 −3.4)		4 (3–5.5)						
GA	Preterm	19 (54.29%)		20 (57.14%)		22 (62.86%)		X2 = 0.5477		0.6254 to 1.443		0.760
	Full term	16 (45.71%)		15 (42.86%)		13 (37.14%)						
Indications	Surgical	19 (55.59%)		23 (65.71%)		20 (57.14%)		X2 = 1.024		0.2363 to 1.6247		0.599
	Medical	16 (45.71%)		12 (34.28%)		15 (48.82%)						

Data is presented as Mean ± SD or frequency (%), *SD* standard deviation, *IQR* interquartile range, *GA* gestational age, preterm defined as below 34 weeks of GA. One way ANOVA was used for comparison of the quantitive data. Chi square test (X^2^) was used for comparison of the categorical data

Statistically significant as *p* value < 0.05, *P*1 *p* value between groups A and B, *P*2 *p* value between groups A and C, *P*3 *p* value between groups B and C

Eighty-three cases CICC inserted into the right IJV compared to the left IJV in 22 cases with all three techniques. The success of cannulation was insignificantly different among the studied groups. Two cases underwent SELDINGER’S technicque with surgical isolation of the vein technique, CICC, then were converted to surgical cutdown due to the tiny diameter of the IJV to canulate it. The technique time was significantly shorter in group C compared to group A and group B (*P* < 0.001, < 0.001) and was significantly shorter in group B compared to group A (*P* < 0.001), using One way ANOVA test followed by Post hoc test (Tukey). The duration of CICC placement from insertion to removal was insignificantly different among the studied groups. Table 2.

**Table 2:** Success of cannulation, technique time (min), and duration of CVC placement (weeks) of the studied groups

		Group A (*n*=35)	Group B (*n*=35)	Group C *(n=35)*	Test of sig	95%CI	*P* value
Success of cannulation	1^st^ trial	22 (62.86%)	25 (71.43%)	28 (80%)	X2=2.520	0.6328 to 1.2238	0.284
	2^nd^ trial	13 (37.14%)	10 (28.57%)	7 (20%)			
Technique time (min)		25 ± 5.8	12.5 ± 1.66	4.4 ± 1.5	F = 292.63	10.456 to 22.644	<0.001*
		*P*1<0.001*, *P*2<0.001*, *P*3<0.001*					
Duration of CVC placement (weeks)		2.86±0.77	3.23±0.81	3.03±0.75	F = 1.988	2.595 to 3.508	0.139

Data is presented as Mean±SD or frequency (%), *CVC *Central venous catheterization, one-way ANOVA was used for comparison of the quantitative data, followed by a post hoc test. Chi square test (X^2^) was used for comparison of the categorical data

*Statistically significant as *p* value <0.05, *P*1 *p* value between groups A and B, *P*2 *p* value between groups A and C, *P*3 *p* value between groups B and C

Post retrieval of CICC line patency by US was significantly higher in groups B&C compared to group A (*P* = 0.003, < 0.001), with no significant difference between groups B&C, using one-way ANOVA test followed by Post hoc test (Tukey). Totally thrombosed IJV was observed in 9 (30%) patients in group A (which was preterm, long time of central line insertion and multiple trials of insertion), 4 (11.11%) patients in group B (which was numerous trials of insertion and coagulative diseases) and 2 (4.08%) patients in group C (which was due to low birth weight and preterm). Totally thrombosed IJV was significantly different among the studied groups (*P* = 0.004), being less common in group C, then group B, while more prevalent in group A, using the Chi square test. No complications were observed among the studied groups. Table 3.

**Table 3:** Preservation of patency of the IJV of the studied groups

		Group A (*n*=35)	Group B (*n*=35)	Group C (*n*=35)	Test of sig	95%CI	*P* value
Patency of IJV	Mean± SD	55.75 ± 23.41	75.0 ± 20.08	81.8 ± 19.7	F =13.05	-31.282 to 5.232	0.004*
	Median (IQR)	50(45-75)	75 (50-100)	75 (75-100)			
	Post hoc	*P*1=0.003*, *P*2<0.001*, *P*3=0.144					
Totally thrombosed IJV		9 (25.75%)	4 (11.43%)	2 (5.7%)	X2= 6.067	0.7636 to 6.6296	0.004*

Data is presented as Mean±SD or frequency (%), SD: standard deviation, IJV: internal jugular vein. One-way ANOVA was used for comparison of the quantitative data, followed by a post hoc test. Chi-square test (X2) was used for comparison of the categorical data.

*Statistically significant as *p* value <0.05, *P*1 *p* value between groups A&B, *P*2 *p* value between groups A&C, *P*3 *p* value between groups B&C. The patent part of the vein’s lumen is measured as a percentage of the total diameter of the vein

**Fig. 7:**
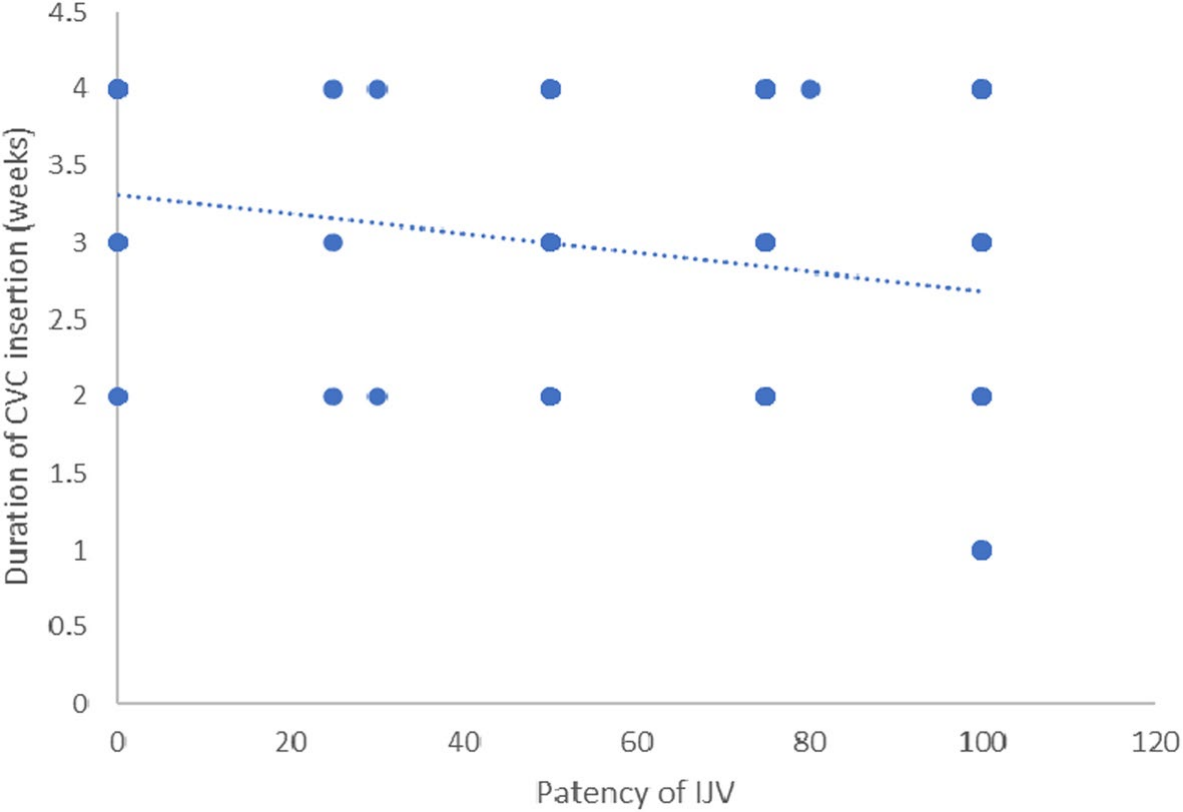
Correlation between patency of IJV and technique time

**Fig. 8:**
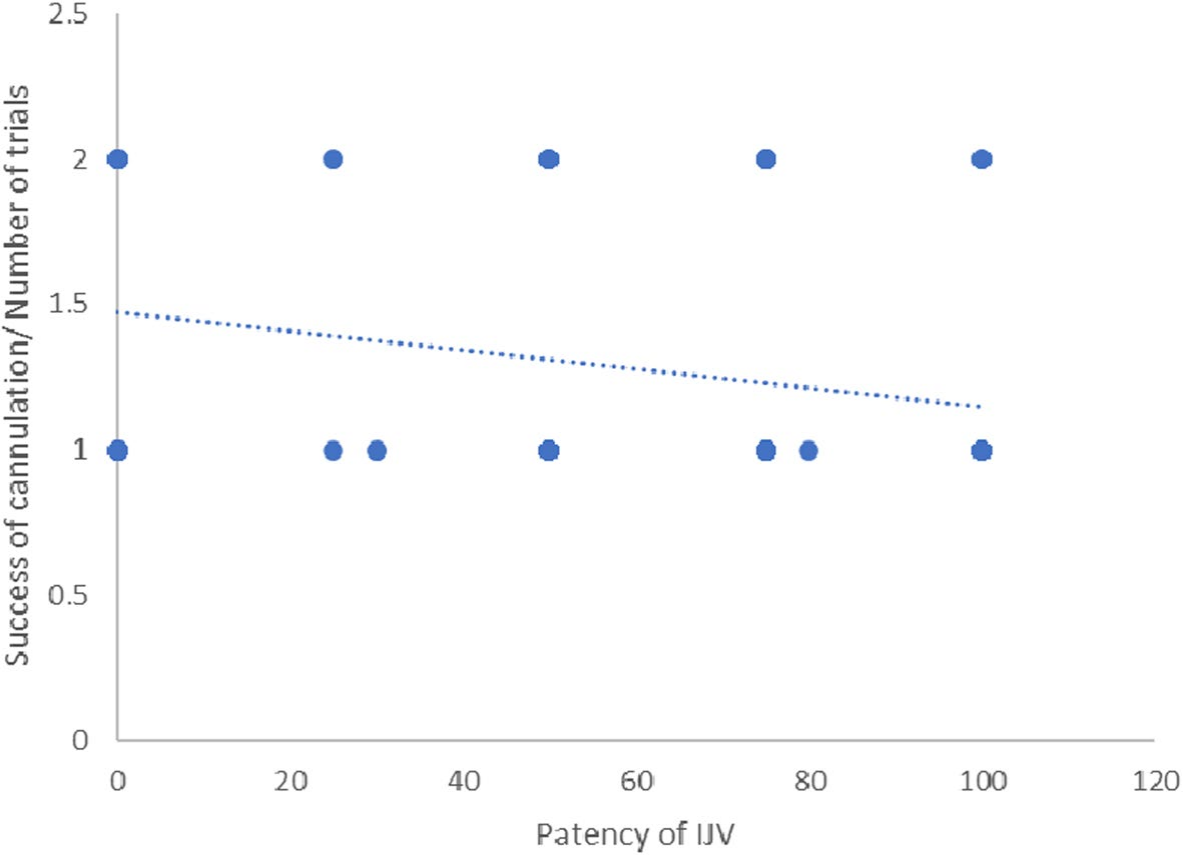
Correlation between patency of IJV and number of trials

There was a significant negative correlation between patency of IJV and duration of CICC placement in all groups (*r* = −0.238, *P* = 0.010) and number of trials to success the cannulation (*r* = −0.252, *P* = 0.006). Figures 7 and 8.

## Discussion

Central venous access devices (CVADs) offer access to the large vessels, thus permitting the administration of drugs contraindicated to be given peripherally [15]. In addition, CVADs are used for longer-term therapy, venous monitoring, and withdrawal of blood samples [6]. The target vein could be punctured directly or by using ultrasound. However, the technique has some degree of threats to failure and complications. In the current study, all cases were of benign pathology with no malignancy. We found that the location of CICC placement was significantly different among the studied groups (*P* < 0.001), either in the NICU, PICU, or operative room, with no significant difference among the studied groups regarding the side, either right or left.

Our results showed no significant difference among the studied groups as regards the success of cannulation. Farhadi et al. [13] showed that the success rate of catheter placement in the ultrasound-guided method was 85.5%, which is in accordance with our results.

Also, Song et al. [16] found that the incidence of successful catheterization on the first attempt was higher in the SELDINGER’S technicque with surgical isolation of the vein group than in the Seldinger’s group. In addition, the number of attempts for guide-wire insertion was lower in the SELDINGER’S technicque with surgical isolation of the vein group than in the Seldinger’s group. However, the guide-wire insertion time in seconds was longer in the SELDINGER’S technicque with surgical isolation of the vein group than in the Seldinger’s group; the total central venous catheterization time was similar in both groups. The difference from our results may be attributed to different patient groups. Schummer et al. [17] and Machata et al., [18] showed that the use of ultrasound increases the success rate of catheterization in the first attempt, reduces the time needed to access the vessel, boosts the overall success rate, and reduces complications, which is going with the results of our current research.

In the present study, we found that the mean operative time is 25 ± 5.8 in group A, versus 12.5 ± 1.66 in group B and 4.4 ± 1.5 in group C. The technique time was significantly shorter in group C when compared to either group A or group B, and was significantly shorter in group B when compared to group A. Shalaby et al. [6] showed a similar result that the operative time of insertion of CICC ranged from 15 to 35 min with a mean of (23.71 ± 3.82). However, Farhadi et al. [13] found that there was no statistically significant difference between the Seldinger’s method and open surgical cutdown (OSC) methods regarding complications. According to their observations, the incidence of thrombosis, infection, and bleeding in Seldinger’s method under ultrasound guidance was less than that of the open approach, but the difference was not significant.

To our knowledge, this is the first study comparing three techniques of insertion of CICCs to IJV in infants.

Our results showed that the preservation of patency of IJV was significantly higher in groups B and C compared to group A (*P* = 0.003, < 0.001), with no significant difference between groups B and C. Total thrombosis of IJV was observed in 9 patients (25.75%) in group A, four patients (11.43%) in group B and two patients (5.7%) in group C. Total thrombosis of IJV was significantly different among the studied groups (*P* = 0.004), being less common in group C, then group B, while more prevalent in group A.

Hosseinpour et al. [19] reported a patency rate of 91% in the open method. Our results of the patency rate in the open group are 96%. However, venous occlusion rates can range from 0 to 15% and 25 to 33% in the ultrasound-guided and open techniques, respectively, in the literature report. This is attributed to the fact that open cut-downs likely lead to more destruction of the vein and induction of endothelial injury [20].

Similar to our results, Machata et al. [18] documented that the implantation of CICCs in children was significantly successful; however, in neonates, the failure rate was high. With regards to catheterization of IJV in children, two meta-analyses of randomized trials found that ultrasound guidance had an advantage over the anatomical landmark approach [21, 22].

Sigaut et al. [23] found that US did not affect the rate of failure of IJV access and the rate of puncture of the carotid artery. We reported similar data for the same point.

In the current study, no complications were reported in any of the studied groups.

However, Shalaby et al. [6] reported some intraoperative complications, such as arrhythmia in 9 cases (7.5%), and blood oozing in 5 cases 4.1%. They had no events of arterial puncture or failure of cannulation. With regard to their postoperative complications, there were 3 cases of Pneumothorax (2.5%), neck hematoma in 2 cases (1.6%), IJV thrombosis in 6 cases (5%), and dislodging of the catheter in 3 cases (2.5%). There were no cases of haemothorax, as they did not insert in the subclavian veins, and they had good control of the internal jugular vein during insertion. Breaking or leaking of the catheter was detected in 2 cases (1.6%), no cases leaked through the tunnel, and 5 cases developed infections (4.1%) that were treated by antibiotics according to culture sensitivity. The new technique saves time on venous dissection, venotomy, and control of bleeding and closure of venotomy. They faced less risk of bleeding as we avoided the vascular dissection and venotomy (which are the main steps of the routine open technique). Postoperatively, this technique minimizes vein thrombosis and gives a chance for reinsertion in the same site over a guide-wire, like the advantage of the closed method; this advantage is lacking in the routine open technique.

Basford et al. [24] in their comparison of complication rates between surgical and radiologic placement, found higher rates of infectious and mechanical complications among surgically placed catheters than among those placed radiologically (47.1% vs 16.7% and 50.0% vs 16.7%, respectively). Mean complications per 1000 days reflected this trend, but just failed to reach significance.

Our study had limitations as

1. Lack of Outcome Assessor Blinding: As noted in the Major Concerns, the apparent lack of blinding for the sonographer assessing IJV patency introduces a significant risk of detection bias and should be acknowledged as a key limitation.
2. Single-Center Study: The study was conducted at a single institution, which may limit the generalizability of the findings to other settings with different patient populations or operator experience levels.
3. Operator Experience: The manuscript does not detail the experience level of the operators performing the different CVC techniques. Variability in operator skill could influence outcomes, particularly technique time and success rates, and represents an unaddressed potential confounder.
4. Short-Term Follow-up: Assessing IJV patency only two weeks post-removal provides limited information on long-term vessel outcomes, which could potentially differ between techniques involving venotomy (Group A) versus puncture (Groups B/C).

## Conclusions

US-guided catheterization of the IJV technique is the best technique as it is a less time-consuming technique with a high first attempt and insertion success rate, with a small number of trials when compared to CICC using either open surgical technique or SELDINGER’S technique with surgical isolation of the vein technique. The following advantageous technique is SELDINGER’S technique with surgical isolation of the vein, which can be chosen when there is no facility to use an ultrasound device.

Therefore, a larger multicenter cohort with a larger sample size is recommended. The use ECG is highly recommended to minimize the need to use X-rays. Replacement of heparinized flushing with normal saline is also recommended. Ultrasound-guided technique is recommended as it allows the safe placement of any central catheter, as it enables visualization of the vein and measurement of its size, and reduces thrombosis and all types of surgical risks.

## Supplementary Information

Supplementary Material 1.

Supplementary Material 2.

## Data Availability

The data supporting the present findings are contained within the manuscript.

## Abbreviations

CICC: Centrally inserted central catheter
IJV: Internal jugular vein
ICUs: Intensive care units
US: Ultrasound
PICCs: Peripherally inserted central venous catheter
NICE: National Institute for Clinical Excellence
2D: Two-dimensional
SD: Standard deviation
IQR: Interquartile range
%: Percentage
CVADs: Central venous access devices
OSC: Open surgical cutdown
