# Supplementary figures and images for: Comparing open surgical, SELDINGER’S technique with surgical isolation of the vein and ultrasound guided techniques for jugular central line insertion in infants: a randomized clinical trial
Source: BMC Surg. 2025 Jul 3;25:280. doi: 10.1186/s12893-025-02988-5 (PMC12442283; doi:10.1186/s12893-025-02988-5)

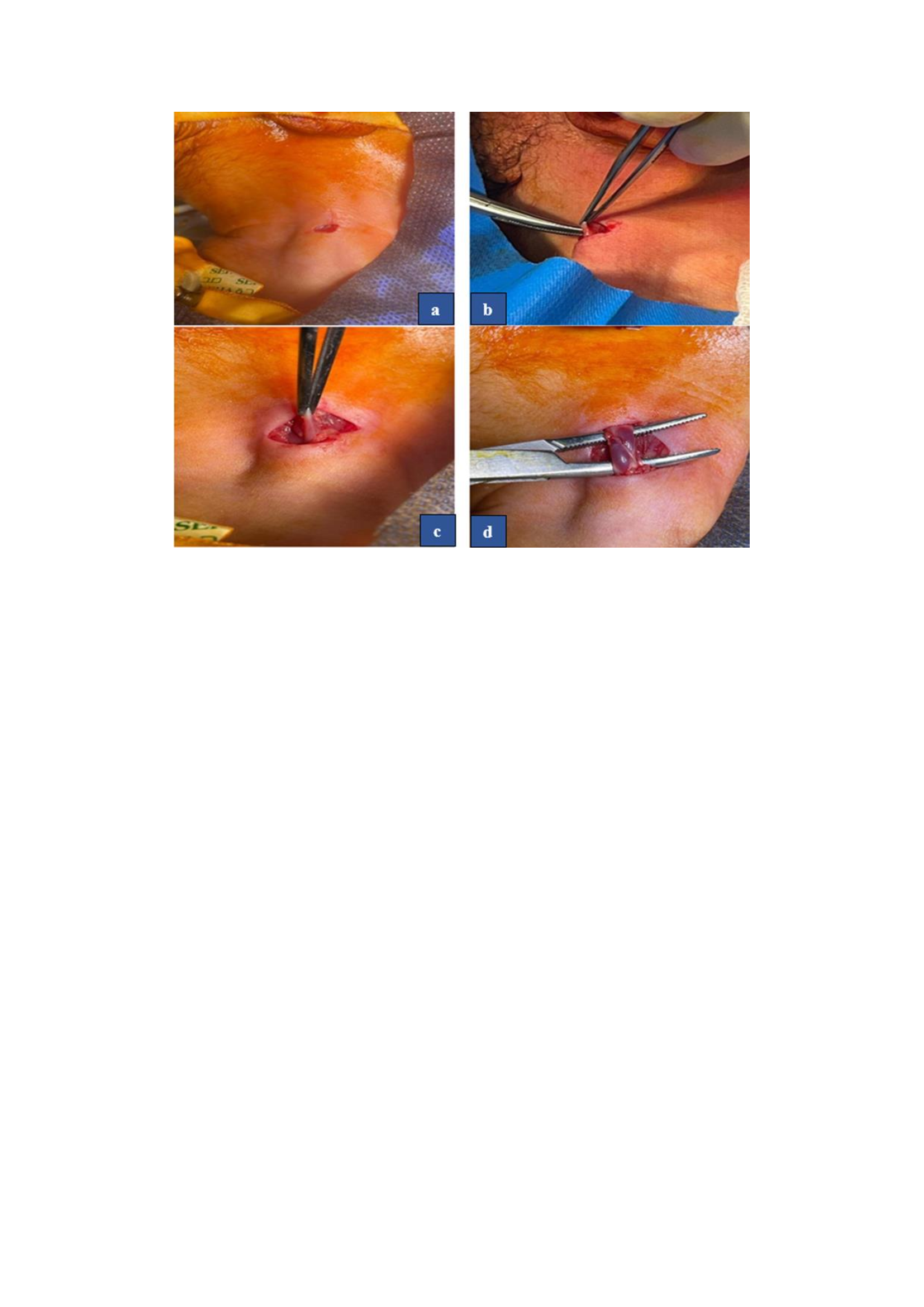

Supplement: Supplementary file 1 — Supplementary Material 1. [file 12893_2025_2988_MOESM1_ESM.zip › 1-Figure 1.tiff]

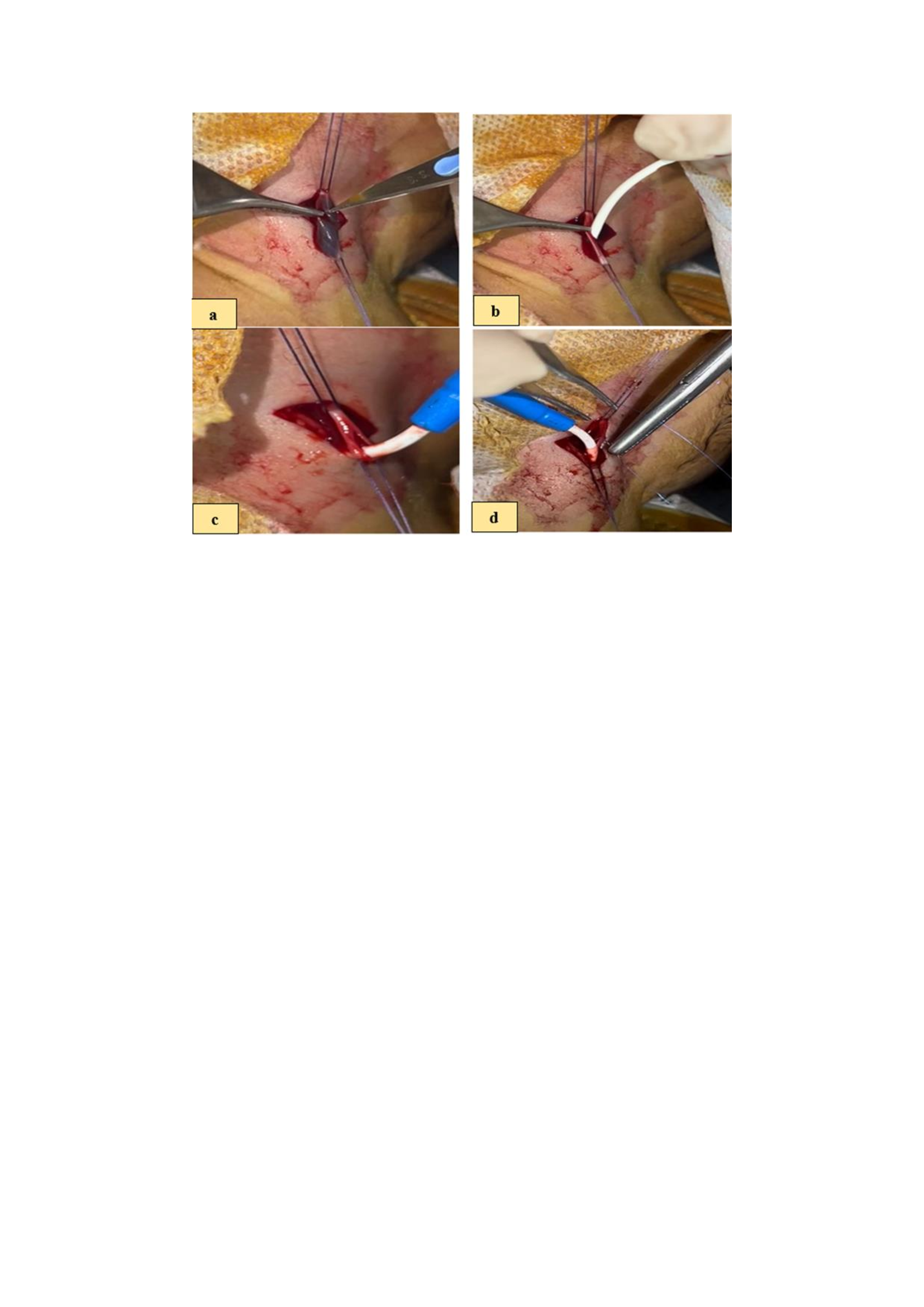

Supplement: Supplementary file 1 — Supplementary Material 1. [file 12893_2025_2988_MOESM1_ESM.zip › 1-Figure 2.tiff]

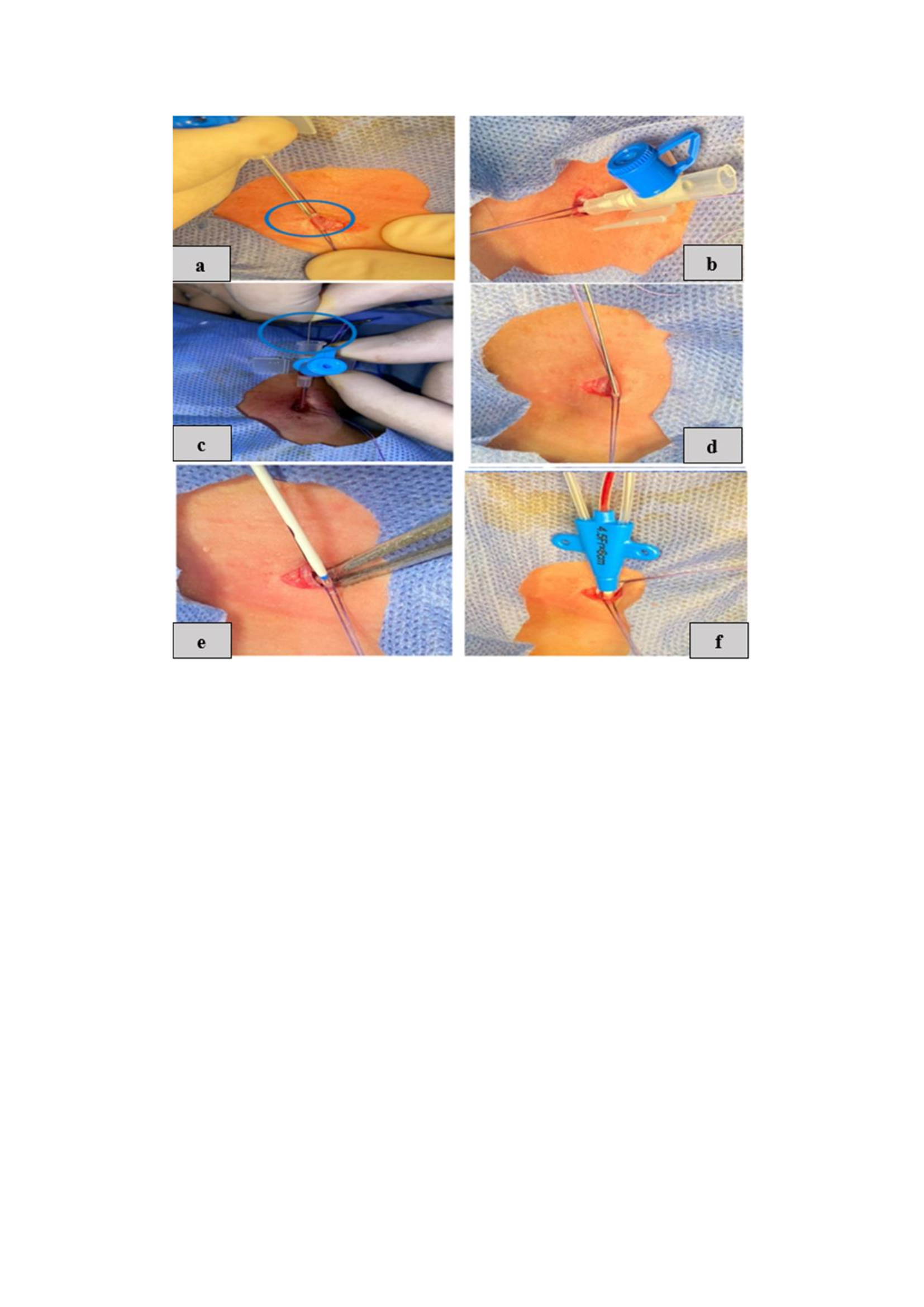

Supplement: Supplementary file 1 — Supplementary Material 1. [file 12893_2025_2988_MOESM1_ESM.zip › 1-Figure 3.tiff]

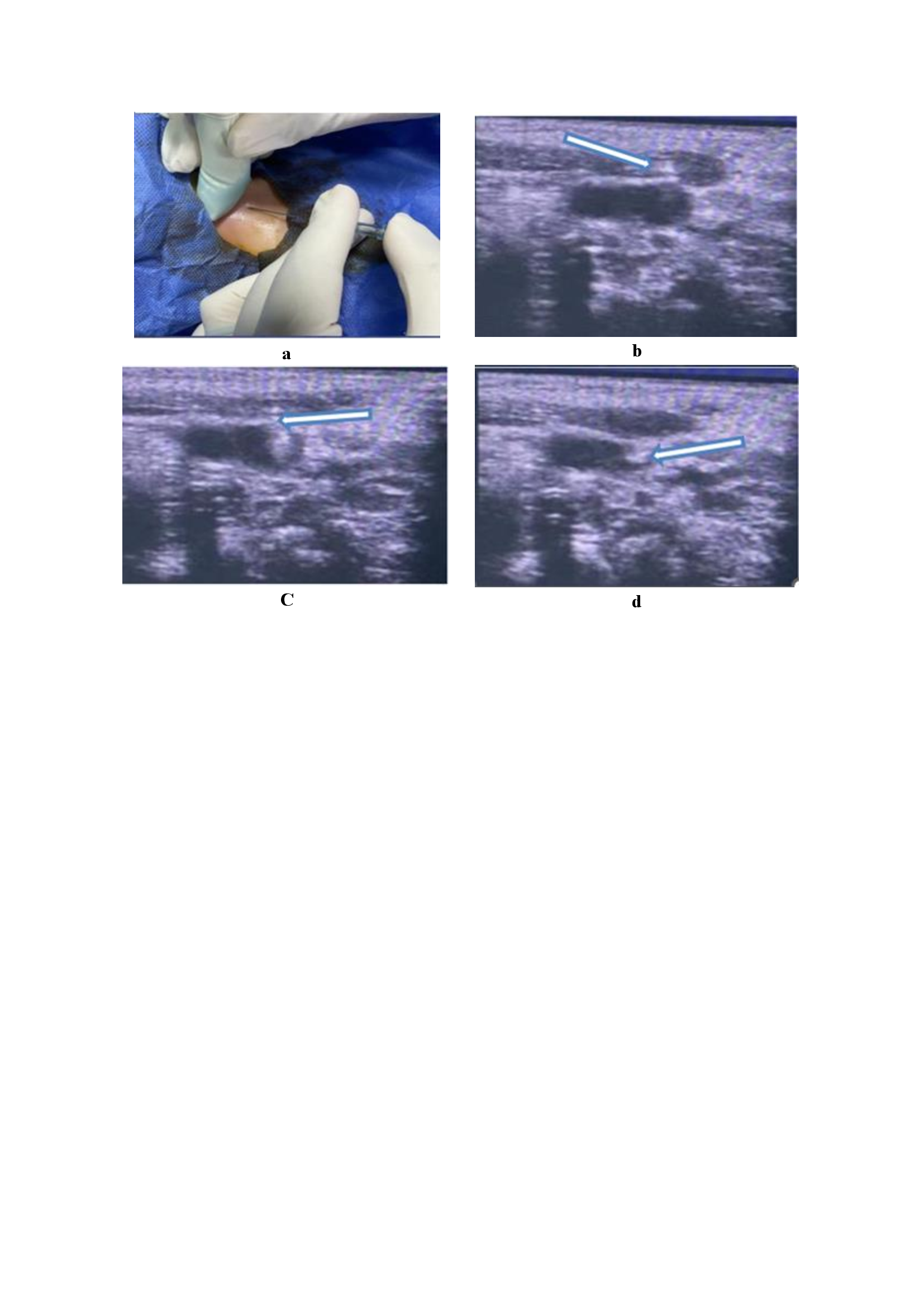

Supplement: Supplementary file 1 — Supplementary Material 1. [file 12893_2025_2988_MOESM1_ESM.zip › 1-Figure 4.tiff]

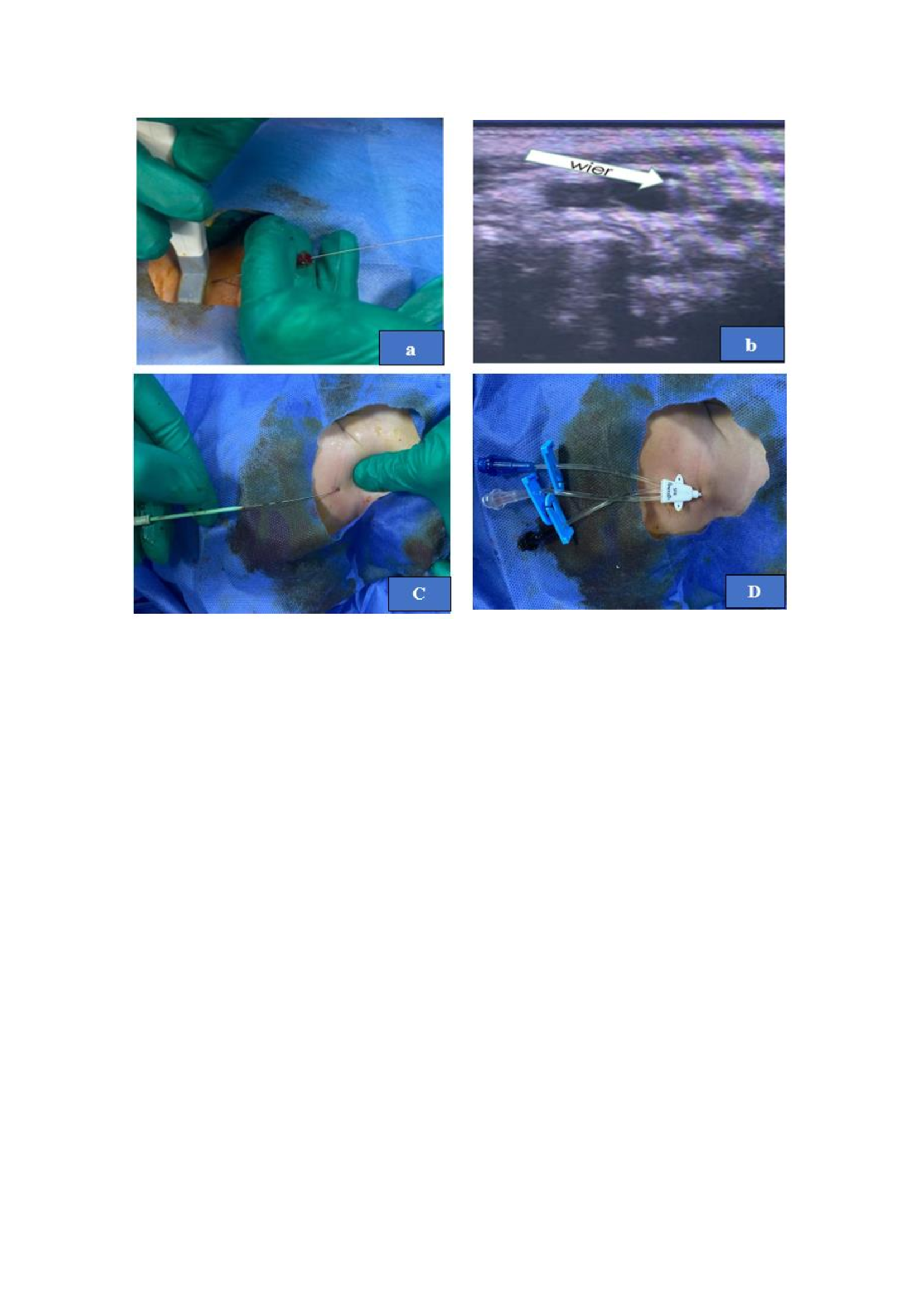

Supplement: Supplementary file 1 — Supplementary Material 1. [file 12893_2025_2988_MOESM1_ESM.zip › 1-Figure 5.tiff]

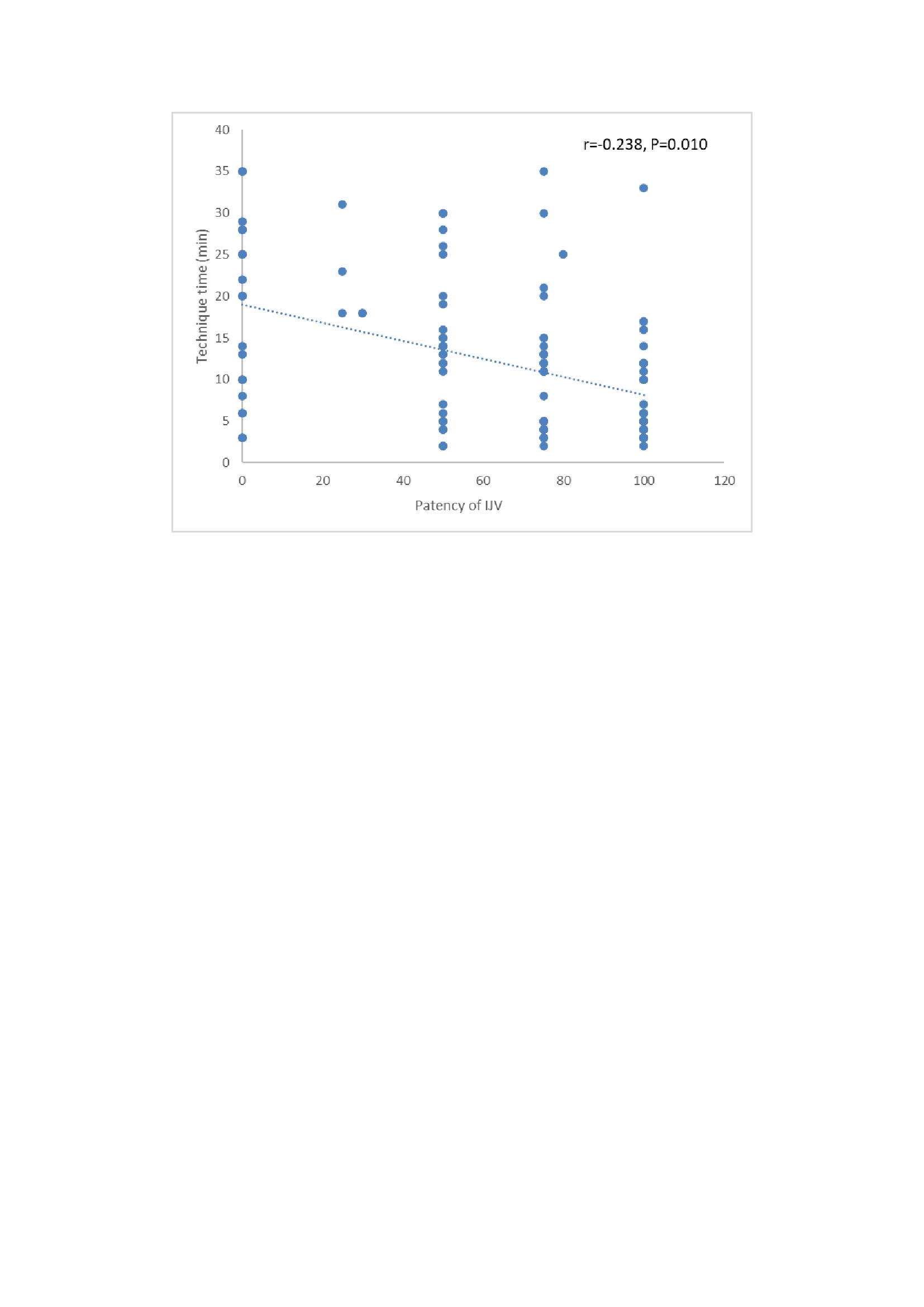

Supplement: Supplementary file 1 — Supplementary Material 1. [file 12893_2025_2988_MOESM1_ESM.zip › 1-Figure 7.tiff]

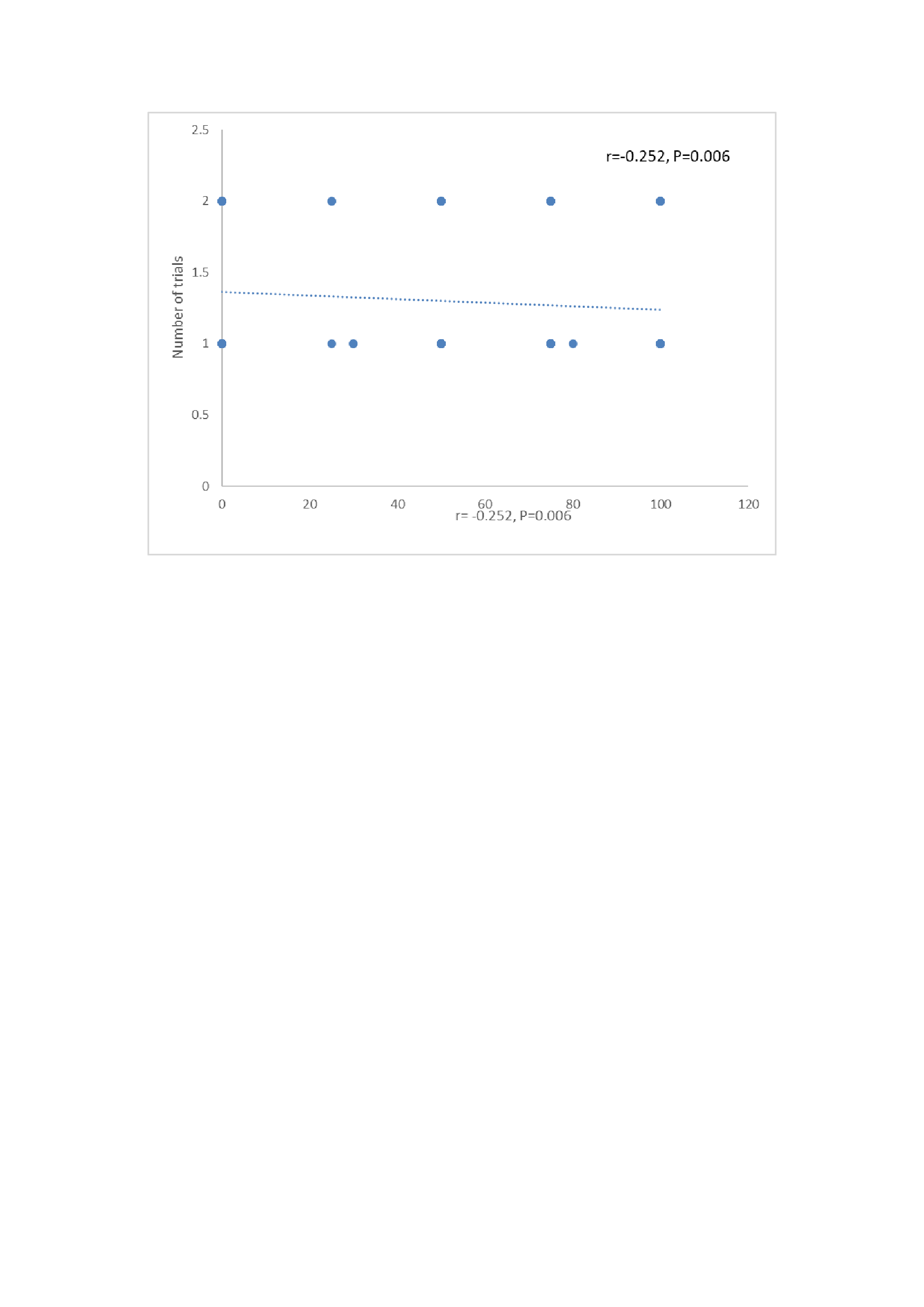

Supplement: Supplementary file 1 — Supplementary Material 1. [file 12893_2025_2988_MOESM1_ESM.zip › 1-Figure 8.tiff]
